# Endoscopic Use of N-Butyl-2-Cyanoacrylate in Refractory Pancreatic Duct Leak and Cystic Duct Leak: Is It Really a Last Resort?

**DOI:** 10.3390/jcm14103362

**Published:** 2025-05-12

**Authors:** Mario Gagliardi, Carlo Soldaini, Mariano Sica, Carmela Abbatiello, Michele Fusco, Federica Fimiano, Giuseppina Pontillo, Elio Donnarumma, Alessandro Puzziello, Claudio Zulli

**Affiliations:** 1 Digestive Endoscopy Unit, San Giovanni di Dio e Ruggi d’Aragona University Hospital, Gaetano Fucito Location, Mercato San Severino, 84131 Salerno, Italy; mariano.sica@sangiovannieruggi.it (M.S.); carme.abbatiello@gmail.com (C.A.); federicafimiano@gmail.com (F.F.); giu.pontillo@gmail.com (G.P.); zulli.claudio@gmail.com (C.Z.); 2 Gastrointestinal Unit, Department of Medicine, Surgery and Dentistry “Scuola Medica Salernitana”, University of Salerno, 84084 Salerno, Italy; carlo.soldaini@outlook.com; 3 A.O.U. San Giovanni di Dio e Ruggi d’Aragona, U.O.C. Clinica Chirurgica e Trapianti di Rene, 35128 Padova, Italy; elio.donn@hotmail.it (E.D.); apuzziello@unisa.it (A.P.); 4 Department of Surgery and Transplants, AOU San Giovanni di Dio and Ruggi d’Aragona, University of Salerno, 84084 Salerno, Italy

**Keywords:** N-butyl-2-cyanoacrylate, pancreatic duct leak, cystic duct leak, endoscopic retrograde cholangiopancreatography

## Abstract

**Background/Objectives**: The management of refractory pancreatic duct (PD) and cystic duct leaks may represent an endoscopic challenge. Standard endoscopic therapy involves pancreatic/biliary sphincterotomy and stenting during endoscopic retrograde cholangiopancreatography (ERCP). After conservative (fasting, parenteral nutrition, and use of somatostatin analogs) or conventional endoscopic treatments fail, a surgical approach is usually required, leading to higher mortality due to several technical complications. Previous evidence of the endoscopic use of N-butyl-2-cyanoacylate (NBCA) for treating pancreaticobiliary leaks is reported, although the evidence is scarce. **Methods**: Six patients with pancreaticobiliary leaks (three IT pancreatic leaks and three Class A sec. Strasberg post-cholecystectomy biliary leaks) refractory to previous treatment were treated with the endoscopic topical application of NBCA. All our patients gave informed consent. The procedures were all performed between December 2017 and February 2025 at a tertiary referral center for biliopancreatic endoscopy. **Results**: Therapeutic success, as shown both endoscopically and radiologically, was 100%, and no procedural complication was reported. In one patient with biliary leak, standard cannulation of the cystic duct stump with the guidewire was unsuccessful, requiring a peroral cholangioscopy (SpyGlass System DSII) to directly visualize the leakage site, allowing a precise closure of the wall defect with NBCA. **Conclusions**: NBCA injection could represent a safe and effective endoscopic option in refractory PD of the tail of the pancreas and cystic duct leaks. Larger and further studies are necessary to confirm these results.

## 1. Introduction

N-butyl cyanoacrylate (NBCA) is a synthetic adhesive that is commonly used in a variety of medical procedures. Cyanoacrylates are adhesives that polymerize when in contact with a weak base, such as blood, bile, and pancreatic fluids. NBCA is commonly used in gastrointestinal endoscopy to treat gastric varices and as a surgical sealing for gastrointestinal and pancreaticobiliary fistulas [1,2]. Furthermore, NBCA is frequently adopted as an embolizing agent for interventional vascular procedures [3]. Pancreatic duct (PD) disruption occurs when the integrity of the PD is lost, mainly as a result of abdominal surgery, trauma, or pancreatitis, leading to various complications such as ascites, fluid collection, fistulas, pseudocysts, and abscesses [4]. Postoperative PD leak, with an incidence rate that can range from 3 to 45%, is a significant issue for pancreatic surgeons [5]. These complications can cause significant morbidity, increased hospital stay, and higher costs. The management of PD leaks includes conservative, endoscopic, or surgical methods. Endoscopic treatment, particularly pancreatic stent placement and sphincterotomy during endoscopic retrograde cholangiopancreatography (ERCP), is preferred for patients who do not respond well to conservative treatment [4].

A postoperative biliary leak occurs when bile escapes outside the biliary tree due to biliary tract injuries during procedures such as laparoscopic cholecystectomy. In most cases, the residual cystic duct is the leakage site, posing significant therapeutic challenges [6]. The management of biliary leaks includes percutaneous biloma drainage, percutaneous transhepatic cholangiography (PTC) with drainage, or ERCP with sphincterotomy, stenting, and nasobiliary drain placement [7].

Overall, endoscopic treatment is a highly effective first-line approach for managing pancreatobiliary leaks, with high success rates, up to 75–90% [8,9,10,11].

On the other hand, pancreaticobiliary leaks refractory to standard endoscopic treatment often require a surgical approach which may result in high morbidity (14–20%) and even mortality (1–20%) [12]. Nowadays, there is no standardized treatment algorithm for refractory pancreaticobiliary leaks.

Previous evidence of the endoscopic use of NBCA for treating pancreaticobiliary leaks is reported, although the evidence is scarce.

In this case series, we report the topical endoscopic use of NBCA as a rescue therapy in patients with PD and cystic duct leak, refractory to a first-line treatment, and a single case of a cholangioscopy-guided application of NBCA for a complex cystic duct leak, refractory to previous endoscopic treatment.

## 2. Materials and Methods

Six consecutive patients with pancreaticobiliary leaks (three PD leaks at the tail of the pancreas and three post-cholecystectomy biliary leaks) refractory to previous treatment were treated with the endoscopic topical application of NBCA (Glubran 2, GEM srl Viareggio, Italy). All our patients gave informed consent. The procedures were all performed between December 2017 and February 2025 at a tertiary referral center for biliopancreatic endoscopy by two expert endoscopists (CZ, MG).

In all patients, conservative treatment or endoscopic sphincterotomies (EST) were already previously performed. All ERCPs were performed with the patient in the supine position and under deep sedation with endotracheal intubation. After the placement of a guidewire in the cystic duct stump or PD, a 5 Fr standard ERCP cannula with a distal radiopaque marker was first flushed with a 5% glucose solution to avoid polymerization in the catheter, then an amount of 1–2 mL of NBCA diluted with contrast medium (ratio 1:1.5–2) was injected topically in the cystic duct or PD under fluoroscopy in the leakage site. In all PD leak patients, the injection 1 mL was sufficient to seal the fistula. On the other hand, for 2/3 patients with cystic duct leak, a total of 2 mL of NBCA was necessary to completely seal the leakage, due to larger size defects. The choice to dilute NBCA with contrast medium was made to directly visualize the amount of “NBCA mixture” applied during fluoroscopy. In all patients with biliary leak, a second guidewire into the common bile duct was placed, over which a plastic stent was deployed to prevent the back leakage of NBCA obstructing the main bile duct.

After the procedure, patients were monitored both clinically and biochemically (CRP and amylase output from the abdominal drainage).

The gradual retraction of percutaneous drainage with evidence of a progressive reduction in the outflow observed 24–48 h after the procedure proved fistula closure in all patients. All patients underwent a scheduled Magnetic Resonance Cholangiopancreatography (MRCP) 14 days after confirming fistula closure.

## 3. Case Reports

### 3.1. Pancreatic Duct Leak

Patient characteristics are reported in Table 1.

**Figure 1:**
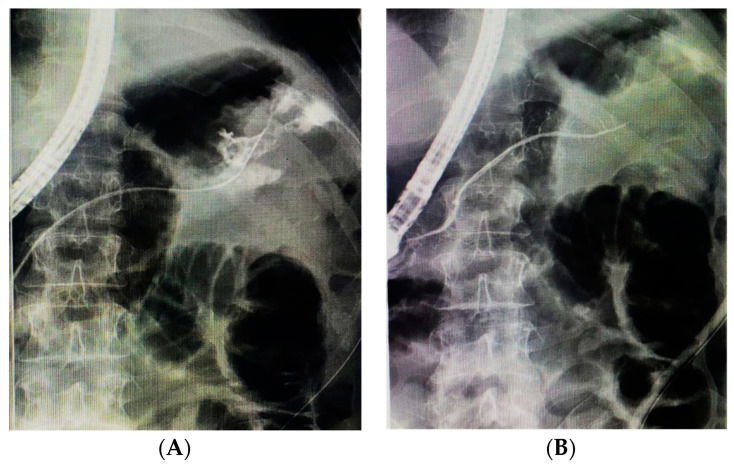
(**A**) Fluoroscopic image of a PD disruption at the tail level with extravasation of contrast medium. (**B**) Evidence of the leak resolution after injection of N-Butyl-2-Cyanoacrylate.

Patient 1. A 51-year-old male was involved in a car accident and sustained severe abdominal trauma. Upon admission, he was hemodynamically unstable and underwent an emergency laparotomy, which revealed a shattered spleen. An open splenectomy was successfully performed. Postoperatively, there was persistent abdominal drainage with high amylase content, raising suspicion of a pancreatic fistula. Further imaging confirmed the presence of fistulous communication between the pancreatic duct (tail) and the peritoneal cavity. An ERCP was performed to manage the pancreatic fistula; pancreatography showed a contrast leak at the tail of the pancreas, without main pancreatic duct narrowing at that site. A pancreatic sphincterotomy was carried out to facilitate drainage and reduce intraductal pressure, and a pancreatic plastic stent (5 Fr) was placed in the PD bridging the leak site. Despite this intervention, the pancreatic fistula persisted as the recurrent output from his abdominal drainage. After one week, given the failure of conservative and standard endoscopic measures, a decision was made to apply cyanoacrylate endoscopically as a salvage therapy (Figure 1), three weeks after the accident (21 days).

Patient 2. A 38-year-old male sustained multiple stab wounds to the abdomen and was brought to the emergency department in hemorrhagic shock. An urgent exploratory laparotomy was performed, revealing a severely injured spleen with active bleeding. Due to the extent of the damage, an open splenectomy was carried out successfully. Postoperatively, the patient developed a persistent high-amylase output from his abdominal drain. Imaging studies confirmed the presence of a pancreatic duct leak, located at the tail of the pancreas. Pancreatography during ERCP showed a contrast leak at the tail of the pancreas with a narrow distal main pancreatic duct that did not permit bridging stenting. In light of this, an endoscopic sphincterotomy and the application of NBCA at the site of the PD leak were performed two weeks after the injury (15 days).

Patient 3. A 63-year-old female involved in an accident at work underwent open splenectomy in a spoke hospital. The surgery was uneventful, and she was initially stable postoperatively. However, in the following days, she developed a persistent pancreatic leak, as indicated by high-amylase output from her surgical drain. The fistula persisted despite conservative management, including dietary modifications and somatostatin analogs. An ERCP was performed; pancreatography showed a contrast leak at the tail of the pancreas, without main pancreatic duct narrowing at that site, so a pancreatic sphincterotomy was carried out to facilitate ductal drainage and a plastic stent (5 Fr) was placed, bridging the leak site. However, the leakage remained unresolved, necessitating an alternative approach; so it was decided to apply NBCA endoscopically, successfully sealing the leak, almost three weeks after the accident (20 days).

### 3.2. Cystic Duct Leak

Patient characteristics are reported in Table 2.

Patients 4 and 5. A 66-year-old female and a 49-year-old male underwent laparoscopic cholecystectomy, in both cases due to complicated cholelithiasis. Even if no intraprocedural complication was reported, patients experienced severe abdominal pain, revealing the presence of a cystic duct leak. An ERCP was performed, with sphincterotomy and the placement of a 10 Fr, 5 cm plastic stent. Despite this endoscopic treatment and the progressive retrieval of the percutaneous abdominal drainage, the bile leak was not resolved, and it was decided to apply NBCA endoscopically, successfully sealing the leak.

**Figure 2:**
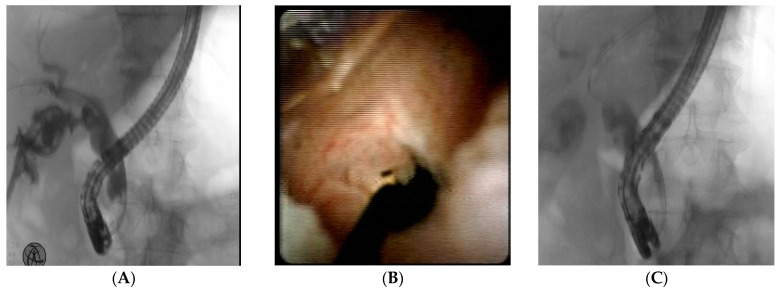
(**A**) Fluoroscopic image of a cystic duct leak with extravasation of contrast medium; (**B**) cholangioscopic view of the leak; (**C**) evidence of the leak resolution after injection of N-Butyl-2-Cyanoacrylate.

Patient 6. A 79-year-old male underwent an open cholecystectomy for acute calculous cholecystitis in a spoke hospital. The procedure was complicated by dense adhesions, requiring careful dissection. On postoperative day 3, the patient developed progressive abdominal pain, fever, and bilious drainage from the surgical site. Laboratory findings revealed elevated white blood cell count and mild hyperbilirubinemia. Abdominal ultrasound and computed tomography (CT) showed a localized fluid collection near the gallbladder fossa. An ERCP confirmed the free extravasation of contrast medium from the cystic duct stump, so a biliary sphincterotomy with the placement of a 10 Fr 5 cm plastic stent was performed. On postoperative day 3, bilious drainage from the surgical site persisted, so a second attempt at ERCP with placement of a biliary Fully Covered Metal Stent (FC-SEMS) was performed. After the intervention, the symptoms were relieved and a progressive retrieval of the percutaneous abdominal drainage was performed. After three days, the patient complained of fever and abdominal pain, with abdominal ultrasonography evidence of fluid collection near the gallbladder fossa, so a percutaneous drainage was established with a high volume bile output. The patient was then referred to our center. During the ERCP, the cystic duct could not be cannulated due to technical difficulties, and for this reason, a peroral cholangioscopy (SpyGlass DS II system) was performed. A 2–3 mm wall defect at the cystic duct stump was detected under direct cholangioscopy; therefore, a selective placement of a guidewire was performed, which was placed over a cannula to apply NBCA. The procedure was well tolerated, and the bile leak ceased immediately. A successive occlusion cholangiogram confirmed the leak resolution (Figure 2).

## 4. Discussion

The endoscopic application of NBCA has been recognized to be useful for sealing biliary and pancreatic tail leaks refractory to standard endoscopic treatment, consisting of sphincterotomy and plastic stenting in the biliary tree or main PD. Currently, although scarce, there is evidence in the literature suggesting that NBCA could play a bigger role in the treatment of pancreaticobiliary fistula in patients not suitable for surgery; however, due to the lack of any controlled trials, its role in therapeutic algorithms has never been standardized.

Pancreatic leaks remain a challenging complication of pancreatic surgery and represent one of the most significant issues for pancreatic surgeons [13]. First-approach conservative treatment consisting of fluid drainage, general support, bowel rest with parenteral nutrition, and somatostatin analogues has a success rate of 50–60% for minor leaks [14]. However, these complex patients are best served by multidisciplinary teams with experienced interventional radiologists, pancreaticobiliary surgeons, and endoscopists [4]. Indeed, endoscopic treatment, particularly ERCP with sphincterotomy and pancreatic stent placement, decreases the transpapillary pressure gradient, facilitating the outflow of pancreatic juice into the duodenum rather than through the duct disruption, allowing the leak to heal [8]. However, in some cases, standard endoscopic techniques may not be sufficient, requiring a surgical approach burdened by high morbidity and mortality [12].

Bile leakage following laparoscopic cholecystectomy (LC) is not uncommon and occurs in approximately 2% of cases [9]. The presence of anatomical variants such as subvesical ducts, running under the Glisson’s capsule of the cystic plate and draining into the anterior right sectoral or hepatic duct, and failing to comply with the ‘Critical View of Safety’ during laparoscopic cholecystectomy, may increase the risk of iatrogenic bile duct injuries, as analyzed by Strasberg et al. almost three decades ago [15].

ERCP plays a key role in the management of biliary leaks by identifying the site of the bile leak and, importantly, allowing internal drainage of the bile. Depending on the grade and location of the leak, the reported success rate of ERCP in this setting ranges from 87.1% to 100% [7]. Bile leaks are classified endoscopically as low-grade, where the leak can only be identified after the opacification of the intrahepatic biliary system, and high-grade, where the leak can be observed before intrahepatic opacification [10]. Endoscopic therapy aims to reduce the transpapillary biliary pressure gradient, thereby facilitating the outflow of bile into the duodenum and allowing spontaneous closure of the leak. This can be achieved by endoscopic sphincterotomy +/− biliary stent (BS) placement [10]. The role of sphincterotomy alone in the management of these patients is currently not well agreed upon. A recent systematic review and meta-analysis reported that EST plus biliary stent placement is more effective than BS alone [11]; this may be related to the inter-endoscopist variation in the completeness of sphincterotomy and post-sphincterotomy edema, which may influence the preferential trans-papillary flow of bile. Baron et al. reported the first case series on the successful use of a covered self-expandable metal stent (CSEMS) for the closure of post-cholecystectomy biliary leak refractory to endoscopic sphincterotomy and plastic stent placement [16]. Kahaleh et al. conducted a pilot study analyzing the effectiveness of the temporary insertion of SEMS in a cohort of 16 patients; in all but 1 patient there was a complete resolution of the leak on imaging [17]. The suggested benefits of SEMS include its larger diameter and longer patency. However, the potential limitations of this approach cannot be taken into account. SEMS could migrate both in and out of the bile duct, with possible adverse events during removal; in addition, the significant cost of a SEMS can limit the widespread use of this approach.

The present study reports a case series of the successful use of NBCA in 5 patients with pancreaticobiliary fistula refractory to the previous treatment and candidates for surgical repair, and a single case (Patient 6) where NBCA was used after a cholangioscopy-guided cannulation of the cystic duct for a complex biliary leak, refractory to previous treatment.

In particular, we reported three cases of refractory pancreatic duct leaks that occurred after open splenectomy for stabbing and trauma; in all patients, conservative treatment, peripancreatic fluid percutaneous drainage, and/or endoscopic therapy with pancreatic stent placement were previously executed, with poor clinical response.

The use of cyanoacrylates, such as NBCA, in pancreatic leaks has emerged in the last few decades [8]. Seewald et al. first published a preliminary study with the successful closure of pancreatic fistula with NBCA in eight patients, with a success rate of 66.6% [18]; consequently, Mutignani et al. reported the use of NBCA for the endoscopic closure of external pancreatic fistulas resistant to conventional endoscopic therapy in four patients [19]. Another case series by Labori et al. reported successful cyanoacrylate/lipiodol injections for persistent pancreatic fistulas, including cases at the level of the pancreatic tail and after distal pancreatectomy [20]. Recently, Sahakian et al. evaluated the feasibility of the endoscopic closure of a pancreatic fistula using a combination of a metal coil and NBCA in a patient with a pancreatic leak refractory to pancreatic stent placement [21]; the rationale was that the metal coil acts as a scaffold for the NBCA, potentially reducing the failure rate associated with glue migration. Mutignani et al. allowed standardization of the endoscopic approach for pancreatic duct disruption [22]; in IT-type leaks, the injection of cyanoacrylate has been proposed if the narrowing of the pancreatic duct at the tail makes the standard approach, with endoscopic sphincterotomy and plastic stent placement bridging the leakage site, impossible.

We described three cases of refractory high-output biliary leaks from the cystic duct stump (Type A sec. Strasberg classification); two occurred after elective LC and one after open cholecystectomy. In these patients, biliary sphincterotomy and biliary stent placement were previously performed as a first-line approach.

The application of NBCA in the cystic stump was first described by Seewald et al. more than 20 years ago. In particular, they reported a small case series of patients with biliary leaks unresponsive to endoscopic drainage; in seven of nine patients, the endoscopic application of NBCA was successful [23]. Since then, few data regarding the use of NBCA in biliary leaks have been reported, with only sporadic case reports [24,25,26].

In Patient 6, the endoscopic use of NBCA was decided after a multidisciplinary case evaluation due to the critical clinical condition of the patient and the massive output of the fistula. During the ERCP, the standard cannulation of the cystic duct stump with the guidewire was unsuccessful; for this reason, a peroral cholangioscopy (SpyGlass System DSII) was performed to directly visualize the leakage site, allowing a precise closure of the wall defect with NBCA.

To the best of our knowledge, this is the only case of the cholangioscopy-guided application of NBCA so far.

NBCA’s proven efficacy, significantly lower cost, and wide availability could make the topical endoscopic application of NBCA preferable to the use of CSEMS as rescue therapy for refractory biliary leaks. Another “pro” to the use of NBCA is its immediate effect, which precludes the need for successive procedures and prolonged hospitalization.

However, there are a few important factors to take into account, such as the potential for adhesive substance migration or a local inflammatory response [2]. Moreover, the potentially life-threatening risks of injecting NBCA are systemic embolism with possible pulmonary embolism, splenic and portal vein thrombosis (which can lead to hepatic decompensation in end-stage liver disease), splenic infarction, and recurrent sepsis due to embolized glue acting as a septic focus. Factors that may increase the risk of embolism include overdiluting the NBCA or injecting too large a volume of NBCA [2].

Considering the above-mentioned risks, NBCA application should be avoided in patients with known hypersensitivity to cyanoacrylate compounds, when there is a high risk associated with applying it in a blood vessel or in settings lacking adequate operator expertise or appropriate imaging guidance, given the potential for serious adverse events and non-target tissue injury.

These risks underscore the need for judicious case selection and operator expertise. Compared to other salvage options such as repeat endoscopic interventions or surgery, NBCA offers a minimally invasive alternative that may reduce morbidity and hospitalization time, particularly in high-risk surgical candidates. However, the absence of prospective comparative data limits the possibility of definitive conclusions regarding its superiority or long-term outcomes. As such, NBCA should be considered a viable adjunct or rescue modality in select patients, pending further validation through controlled studies.

Furthermore, the technique’s broad use is restricted by the absence of standardized methods, necessitating additional validation through prospective studies involving a bigger sample. In fact, an important limitation of our study is the small sample size of patients, although there are no studies in the current literature involving the use of NBCA in a significant number of patients. Lastly, it is noteworthy that in this case series, all the procedures were performed by two experienced endoscopists in a tertiary referral center for biliopancreatic endoscopy, so the safety and the feasibility of NBCA endoscopic application for biliopancreatic leaks in a lower volume center is yet to be explored.

## 5. Conclusions

In conclusion, the topical application of NBCA seems to be a promising endoscopic therapy option for pancreaticobiliary leaks refractory to standard therapy. An accurate diagnosis and a multidisciplinary approach are essential to optimize the outcome of the treatment. Considering the positive outcomes, additional clinical trials, especially prospective comparative studies against established endoscopic and surgical interventions, are still required to evaluate its safety and effectiveness on a broad scale, and thus to further support its application in clinical practice.

## Figures and Tables

**Table 1:** Characteristics of patients with PD disruption treated with NBCA injection.

	Patient 1	Patient 2	Patient 3
**Age**	51	38	63
**Sex**	M	M	F
**Etiology**	Open splenectomy (car accident)	Open splenectomy (stab wounds)	Open splenectomy (accident at work)
**Location**	Tail (IT)	Tail (IT)	Tail (IT)
**Days after the previous unsuccessful** **Endoscopic treatment**	14	N/A	10
**NBCA injected**	1 mL	1 mL	1 mL
**Outcome**	Healing of the fistula	Healing of the fistula	Healing of the fistula
**Complications**	None	None	None

NBCA = N-Butyl-2-Cyanoacrylate; N/A = not available.

**Table 2:** Characteristics of patients with cystic duct leak treated with NBCA injection.

	Patient 4	Patient 5	Patient 6
**Age**	66	49	79
**Sex**	F	M	M
**Etiology**	Laparoscopic cholecystectomy	Laparoscopic cholecystectomy	Open cholecystectomy
**Location**	Cystic Duct (Type A sec. Strasberg)	Cystic Duct (Type A sec. Strasberg)	Cystic Duct (Type A sec. Strasberg)
**Days after previous unsuccessful** **endoscopic treatment**	10	6	3
**NBCA injected (mL)**	1 mL	2 mL	2 mL
**Outcome**	Healing of the fistula	Healing of the fistula	Healing of the fistula
**Complications**	None	None	None

NBCA = N-Butyl-2-Cyanoacrylate.

## Data Availability

The original contributions presented in this study are included in the article. Further inquiries can be directed to the corresponding author.

## Abbreviations

The following abbreviations are used in this manuscript:

NBCA	N-Butyl-2-Cyanoacrylate
ERCP	Endoscopic Retrograde Cholangiopancreatography
PD	Pancreatic duct
LC	Laparoscopic cholecystectomy
EST	Endoscopic sphincterotomy
